# Supplementary material for: Systemic neurotransmitter responses to clinically approved and experimental neuropsychiatric drugs
Source: Nat Commun. 2018 Nov 8;9:4699. doi: 10.1038/s41467-018-07239-1 (PMC6224407; doi:10.1038/s41467-018-07239-1)
Supplement: Supplementary file 1 — Supplementary Information [file 41467_2018_7239_MOESM1_ESM.pdf]

Supplementary Information

Systemic neurotransmitter responses to clinically approved and experimental neuropsychiatric drugs

Noori et al.

Supplementary Methods

KEYWORDS

The online portal of the National Library of Medicine [http://www.ncbi.nlm.nih.gov/pubmed/] including PubMed, PubMed Central and MEDLINE was used as the platform for literature research. A systematic screening of the original research articles published until January 2016 was performed based on the 16,308 keyword combinations: *rat (AND) microdialysis (AND) (brain region (OR) neurotransmitter (OR) metabolite (OR) neuropeptide) (AND) (drug (OR) antidepressant (OR) anxiolytic (OR) psychostimulant (OR) sedative (OR) hypnotic (OR) antipsychotic (OR) neuroleptic).*

Thereby, the brain regions/subregions were defined as: *Main olfactory bulb; Accessory olfactory bulb; Infralimbic cortex; Prelimbic cortex; Anterior cingulate cortex; Lateral prefrontal cortex; Insular cortex, pars anterior; Insular cortex, pars posterior; Nucleus accumbens, shell; Nucleus accubens, core; Caudate Putamen; Lateral septal nucleus; Medial septal nucleus; Nucleus of the diagonal band (horizontal and vertical limb); Septofimbrial nucleus; Triangular septal nucleus; Bed nucleus of the stria terminalis, transverse nucleus; Bed nucleus of the stria terminalis, dorsomedial nucleus; Bed nucleus of the stria terminalis, dorsolateral nucleus; Bed nucleus of the stria terminalis, juxtacapsular nucleus; Bed nucleus of the stria terminalis, supracapsular nucleus; Bed nucleus of the stria terminalis, magnocellular nucleus; Bed nucleus of the stria terminalis, principal nucleus; Bed nucleus of the stria terminalis, ventrolateral nucleus; Bed nucleus of the stria terminalis, ventromedial nucleus; Bed nucleus of the stria terminalis, anterolateral nucleus; Bed nucleus of the stria terminalis, fusiform nucleus; Bed nucleus of the stria terminalis, interfascicular nucleus; Bed nucleus of the stria terminalis, rhomboid nucleus; Bed nucleus of the stria terminalis, anteromedial nucleus; Bed nucleus of the stria terminalis, anterodorsal area; Bed nucleus of the stria terminalis, anteroventral area; Bed nucleus of the stria terminalis, ventral nucleus; Bed nucleus of the stria terminalis, oval nucleus; Bed nucleus of the stria terminalis, posteromedial nucleus; Bed nucleus of the stria terminalus, posterolateral nucleus; Bed nucleus of the stria terminalis, preoptic nucleus; Globus pallidus; Ventral pallidum; Entopeduncular nucleus; Anterior hypothalamic nucleus; Lateral preoptic nucleus; Medial preoptic nucleus; Paraventricular hypothalamic nucleus; Posterior hypothalamic nucleus; Premammillary nucleus (ventral and dorsal); Suprachiasmatic nucleus; Tuberomammillary nucleus; Arcuate nucleus;*

Dorsomedial hypothalamic nucleus; Lateral hypothalamus; Mammillary nucleus (medial and lateral); Periventricular hypothalamic nucleus; Supramammillary nucleus; Supraoptic nucleus; Ventromedial hypothalamic nucleus; Central nucleus of amygdala; Medial nucleus of amygdala; Lateral nucleus of amygdala; Basolateral nucleus of amygdala; Basomedial nucleus of amygdala; Basal nucleus of amygdala; Accessory basal nucleus of amygdala; Cortical nuclei of amygdala (anterior and posterior); Posterior nucleus of amygdala; Nucleus of the lateral olfactory tract; Basal nucleus of the accessory olfactory tract; Medial Habenula; Lateral Habenula; Presubiculum; Postsubiculum; Parasubiculum; Subiculum; Entorhinal cortex; Dentate Gyrus; CA1; CA2; CA3; Perirhinal cortex; Postrhinal cortex; Anteromedial nucleus of thalamus; Anteroventral nucleus of thalamus; Central lateral nucleus of thalamus; Central medial nucleus of thalamus; Intermediodorsal nucleus of thalamus; Lateral geniculate nucleus of thalamus; Medial geniculate nucleus of thalamus; Nucleus reuniens; Paracentral nucleus of thalamus; Parafascicular nucleus of thalamus; Parataenial nucleus of thalamus; Paraventricular nucleus of thalamus; Reticular nucleus of thalamus; Ventral lateral nucleus of thalamus; Ventral medial nucleus of thalamus; Anterodorsal nucleus of thalamus; Interanteromedial nucleus of thalamus; Laterodorsal nucleus of thalamus; Lateroposterior nucleus of thalamus; Mediodorsal nucleus of thalamus; Rhomboid nucleus of thalamus; Subparafascicular nucleus of thalamus; Posterior thalamus; Nucleus submedius; Anterolateral Nucleus Of The Thalamus; Subthalamic Nucleus; Substantia nigra, pars reticulate; Substantia nigra, pars compacta; Substantia nigra, pars lateralis; Rostral linear nucleus; Caudal linear nucleus; Paranigral nucleus; Parabrachial nucleus pigmentosus; Interfascicular nucleus; Dorsal raphe nucleus; Medial raphe nucleus; Nucleus raphe magnus; Nucleus raphe pontis; Nucleus raphe pallidus; Nucleus raphe obscurus; locus coeruleus; Lateral parabarchial nucleus; Medial parabrachial nucleus; Kölliker-Fuse nucleus; Pontine nuclei. Furthermore, the keywords were varied by the anatomical positioning terms and their combinations (e.g. dorsal, lateral and dorsolateral) in order to increase the robustness of the data mining step with respect to anatomical nomenclature.

Furthermore, the keywords neurotransmitter (OR) metabolite (OR) neuropeptide were used in this form as well as replaced with:

2,3-DHBA; 2,5-DHBA; 3-HANA; 3-methoxytyramine; 5-HIAA; ACh; Adrenaline; ALA; Aldosterone; Ammonia; ANA; Ascorbic acid; ASP; CAMP; CCK-8; CCKLM; CGMP;

Cholecystokinin; Choline; Citrulline; Corticosteron; Corticotropin-Releasing Hormone/Factor (CRH and/or CRF); Dopamine (DA); DOPA; DOPAC; DOPEG; Dynorphin; Endorphin (beta-Endorphin); Enkephalin (met-Enkephalin and/or leu-Enkephalin); Epinephrine; GABA; Glutamine (GLN); Glutamate (GLU); Glycine (GLY); Glycerol; Histamine (Hist); HVA; Kyna; Lactate; MHPG; Noradrenaline and/or Norepinephrine (NA and/or NE); Neurotensin; Nitrogen (di-, tri-)oxide (NO, NO2 and NO3); Oxytocin; PTO2; Quin; Serine (SER); Serotonin (5-HT); Somatostatin; Substance P; Taurine, Threonine; TRP; Tyrosine (TYR); Uric Acid; Vasopressin.

List of abbreviations

17	6-(3,4-Dichlorophenyl)-1-[(Methyloxy)methyl]-3-azabicyclo[4.1.0]heptane
8b	N-(4-Ethylamino-phenyl)-guanidine
17b	N-(5,6,7,8-Tetrahydronaphthalen-2-yl)guanidine
20b	4,4-Di(2-imidazolidinylimino)-1,2-diphenylethane
18b	1-(2-Imidazolidinylimino)-4-(N,Ndimethyl) benzene
26b	1-(2-imidazolidinylimino)-3,4-(methylenedioxy)benzene
26a	(3R)-3-{cyclobutyl[3-(5-fluoro-1H-indol-3-yl)propyl]amino}-8-fluorochromane-5-carboxamide
8a	3-[(1S,3R)-3-Dimethylaminocyclopentyl]-1H-indole-5-carbonitrile
9a	[(1R,3S)-3-(5-Fluoro-1H-indol-3-yl)-cyclopentyl]-dimethylamine
12a	trans-2-(5-Cyano-1H-indol-3-yl)-1-(N,N-dimethylaminomethyl)cyclopropane
8i	3-{4-[4-(2-Oxo-2H-1-benzopyran-6-yl)-1-piperazinyl]-butyl}-indol-5-carbonitrile
8h	Ethyl (6-{4-[4-(5-cyano-1H-indol-3-yl)-butyl]-piperazin-1-yl}-2-oxo-2H-chromen-3-yl)-carbamate
8k	N-(6-{4-[4-(5-Cyano-1H-indol-3-yl)-butyl]-piperazin-1-yl}-2-oxo-2H-chromen-3-yl)-acetamide
8f	3-{4-[4-(3-Amino-2-oxo-2H-chromen-6-yl)-piperazin-1-yl]-butyl}-1H-indole-5-carbonitrile
D-473	((2S,4R,5R)-2-(bis(4-fluorophenyl)methyl)-5-((4-methoxybenzyl)amino)tetrahydro-2H-pyran-4-ol)
3-HANA	3-Hydroxyanthranilic acid
3-MT	3-methoxytyramine
5-HIAA	*5-Hydroxyindoleacetic acid*
5-HT	5-hydroxytryptamine
ACh	Acetylcholine
ALA	Alanine
ALDO	Aldosterone
ANA	Anthranilic acid
ASP	Aspartic acid
CAMP	cyclic AMP
CCK-8	Cholecystokinin
CCKlm	Cholecystokinin
cGMP	cyclic GMP

CCK	Cholecystokinin
Chl	Choline
CIT	Citrulline
COR	Corticosterone
CRH	Corticotropin-releasing hormone
DA	Dopamine
DOPA	3,4-dihydroxyphenylalanine
DOPAC	3,4-Dihydroxyphenylacetic acid
DOPEG	Dihydroxyphenylethylene glycol
Dyn	Dynorphin
End	Endorphin
Enk	Enkephalin
GABA	*gamma*-Aminobutyric acid
GLN	Glutamine
GLU	Glutamate
GLC	Glucose
GLY	Glycine
Gro	Glycerol
Hist	Histamine
HVA	Homovanillic acid
KYNA	Kynurenic acid
LAC	Lactic acid
Leu-Enk	Leu-Enkephalin
Met-Enk	Met-Enkephalin
MHPG	3-Methoxy-4-hydroxyphenylglycol
NA	Noradrenaline
NTS	Neurotensin
NO	Nitric Oxides
NO2	Nitric Oxides
NO3	Nitric Oxides
Oxt	Oxytocin
PRL	Prolactin
PtO2	Platinum Oxide
Quin	Quinic Acid
SER	Serine
GHIH	Somatostatin
SP	Substance P
Tau	Taurine
Thr	Threonine
TRP	Tryptophan
TYR	Tyrosine
URI	Uric Acid
ADH	Vasopressin

Supplementary Figures

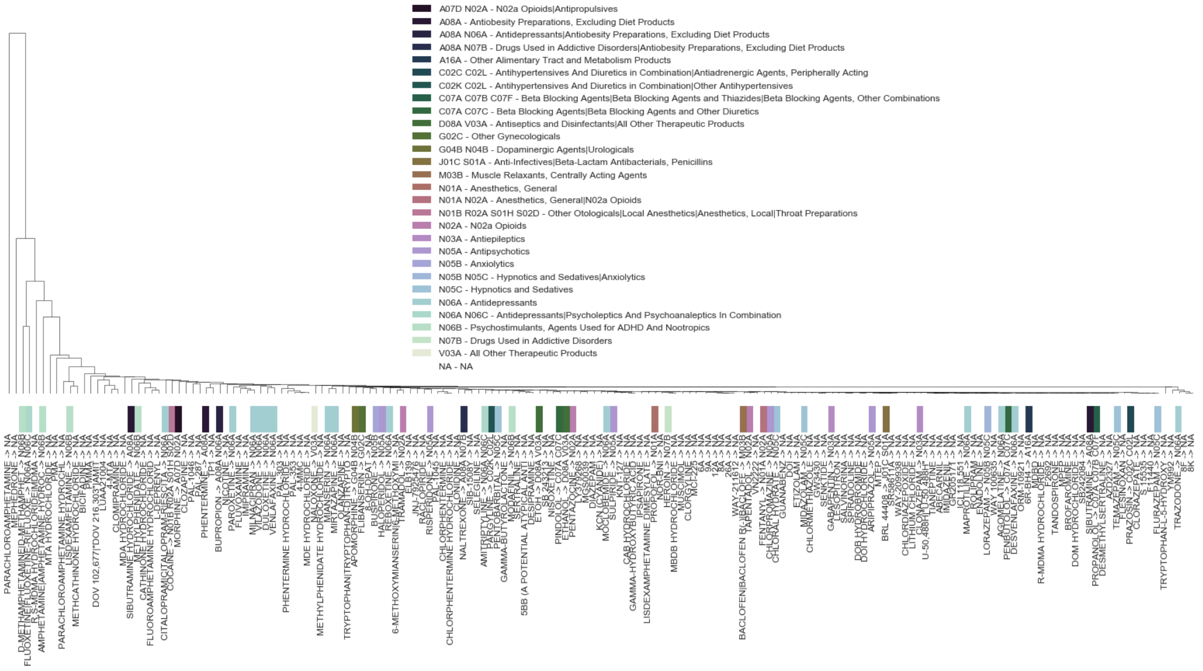

Supplementary Figure 1. Complete clustering compound activity across brain regions and neurotransmitters highlights that the historical classification of active ingredients of drugs (ATC codes) are not exhaustive. It can be seen that there is little correlation between compound activity and codes which hence indicate that the current classification system is not exhaustive or complete. ATC codes are color-coded.

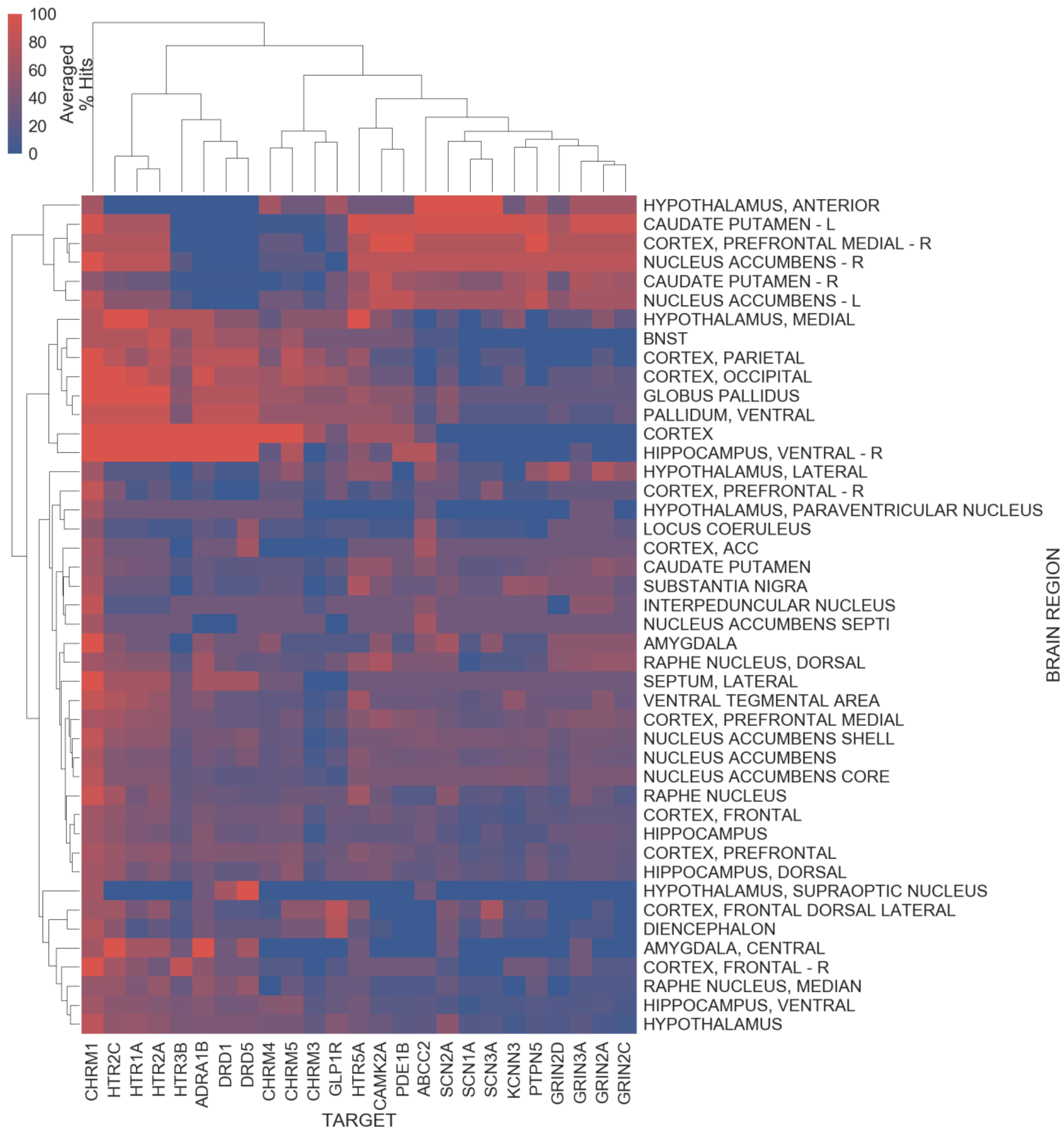

Supplementary Figure 2. Relating targets at different brain regions. We calculated the proportion of predicted protein targets (enriched targets) for each of the drugs active at a neurotransmitter and brain region tuple in the same manner to Figure 2, although the predictions were grouped by and averaged across brain regions. Results from this graph indicate which protein targets are more likely modulated by drugs active at within a given brain region. There are clusters toward the top right hand corner of the heat map (NMDA receptor subunits within the mesocorticlimbic system – NMDA receptors are prime targets of many compounds) and the more left centre of the plot, serotonergic receptors with the cortex and ventral hippocampus – e.g. 5HTR1A autoreceptor is a main target of antidepressant drugs). Non-obvious clusters (without literature-based evidence) in brain regions may reveal new functional connectivity of regions and region-dependent drug target sites..

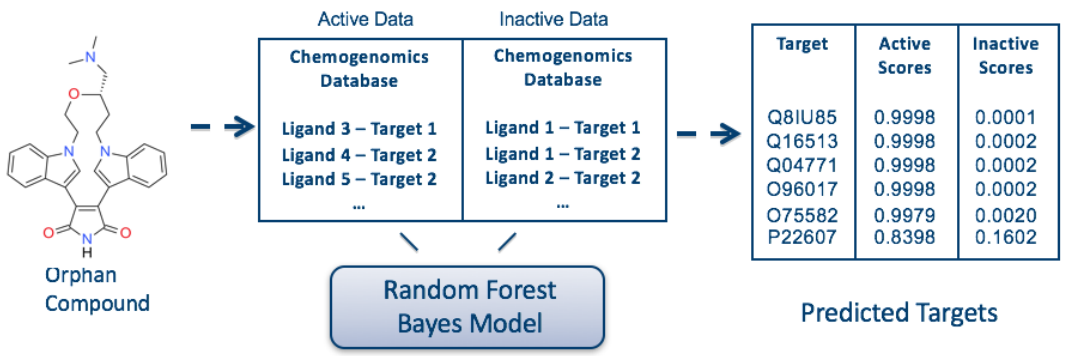

Supplementary Figure 3. Flowchart of the *in silico* target deconvolution protocol. In this procedure, compounds are annotated with their predicted targets using a Random Forest (RF) trained on the active and inactive compounds per target. The models output a probability for the likelihood that an orphan compound will bind to a target based on the chemical similarity of existing ligands. The models are scaled using Platt Scaling so that the probabilities are calibrated to reflect the anticipated true positive rate. We have employed a threshold of 0.8 for use in this study which represents a high confidence (20% error rate allowed) in the predicted targets.

Supplementary Tables

Supplementary Table 1. ATC code classification for the drugs in the database.

ATC CODE	Name
A04A	Antiemetics And Antinauseants
A07D	Antipropulsives
A08A	Antiobesity Preparations, Excluding Diet Products
A16A	Other Alimentary Tract and Metabolism Products
C02A	Antiadrenergic Agents, Centrally Acting
C02C	Antiadrenergic Agents, Peripherally Acting
C02K	Other Antihypertensives
C02L	Antihypertensives And Diuretics in Combination
C07A	Beta Blocking Agents
C07B	Beta Blocking Agents and Thiazides
C07C	Beta Blocking Agents and Other Diuretics
C07F	Beta Blocking Agents, Other Combinations
D08A	Antiseptics and Disinfectants
G02C	Other Gynecologicals
G03D	Progestogens
G03F	Progestogens and Estrogens In Combination
G04B	Urologicals
J01C	Beta-Lactam Antibacterials, Penicillins
M03B	Muscle Relaxants, Centrally Acting Agents
N01A	Anesthetics, General
N01B	Anesthetics, Local
N02A	N02a Opioids
N03A	Antiepileptics
N04B	Dopaminergic Agents
N05A	Antipsychotics
N05B	Anxiolytics
N05C	Hypnotics and Sedatives
N06A	Antidepressants
N06B	Psychostimulants, Agents Used for ADHD And Nootropics
N06C	Psycholeptics And Psychoanaleptics In Combination
N07B	Drugs Used in Addictive Disorders
R02A	Throat Preparations
S01A	Anti-Infectives

S01E	Antiglaucoma Preparations and Miotics
S01H	Local Anesthetics
S02D	Other Otologicals
V03A	All Other Therapeutic Products

Supplementary Table 2. Brain ontology for visualisation purposes.

Brain Regions Without Ontology	Brain Regions With Ontology
BNST	BNST
adrenal gland	adrenal gland
amygdala	amygdala
amygdala - R	caudate putamen
amygdala, BL	cerebellum
amygdala, BL - L	cisterna magna
amygdala, central	cortex, ACC
amygdala, central - L	cortex, PCC
caudate putamen	cortex, cingulate
caudate putamen - L	cortex, entorhinal
caudate putamen - R	cortex, frontal
cerebellum	cortex, frontoparietal
cerebellum - R	cortex, occipital
cisterna magna	cortex, orbito-frontal
cortex	cortex, parietal
cortex, ACC	cortex, perirhinal
cortex, PCC	cortex, prefrontal
cortex, cingulate	cortex, prelimbic
cortex, entorhinal	cortex, primary motor
cortex, frontal	cortex, somatosensory
cortex, frontal - L	cortex, temporal medial
cortex, frontal - R	corpus callosum
cortex, frontal dorsal lateral	diencephalon
cortex, frontal medial	entopenduncular nucleus
cortex, frontal orbital	frontal parenchyma
cortex, frontoparietal - L	globus pallidus
cortex, frontoparietal - R	hippocampus
cortex, occipital	hypothalamus
cortex, orbito-frontal	interpeduncular nucleus
cortex, parietal	locus coeruleus
cortex, perirhinal	medulla
cortex, prefrontal	nucleus accumbens
cortex, prefrontal - L	pallidum, ventral
cortex, prefrontal - R	periaqueductal
cortex, prefrontal medial	pituitary gland
cortex, prefrontal medial - L	raphe nucleus
cortex, prefrontal medial - R	reticular formation
cortex, prelimbic	septum
cortex, primary motor	substantia innominata
cortex, somatosensory	substantia nigra
cortex, temporal medial	thalamus
crus of corpus callosum	ventral tegmental area
diencephalon	ventricle, lateral
entopenduncular nucleus	
frontal parenchyma	
globus pallidus	

globus pallidus - L	
hippocampus	
hippocampus - L	
hippocampus - R	
hippocampus, ca1	
hippocampus, ca3 - R	
hippocampus, dentate gyrus	
hippocampus, dentate gyrus-ca 1	
hippocampus, dorsal	
hippocampus, dorsal - L	
hippocampus, mid-ventral	
hippocampus, posterior	
hippocampus, ventral	
hippocampus, ventral - R	
hypothalamus	
hypothalamus, anterior	
hypothalamus, anterior - R	
hypothalamus, anterior lateral	
hypothalamus, lateral	
hypothalamus, medial	
hypothalamus, paraventricular nucleus	
hypothalamus, periventricular nucleus	
hypothalamus, preoptic anterior	
hypothalamus, preoptic area	
hypothalamus, preoptic area anterior	
hypothalamus, preoptic area medial	
hypothalamus, supraoptic nucleus	
hypothalamus, supraoptic nucleus - R	
hypothalamus, ventromedial	
interpeduncular nucleus	
locus coeruleus	
locus coeruleus - R	
medulla, rostral ventrolateral	
medulla, rostral ventromedial	
nucleus accumbens	
nucleus accumbens shell	
nucleus accumbens - L	
nucleus accumbens - R	
nucleus accumbens core	
nucleus accumbens core - L	
nucleus accumbens core - R	
nucleus accumbens septi	
nucleus accumbens shell	
nucleus accumbens shell - L	
nucleus accumbens shell - R	
pallidum, ventral	
periaqueductal gray	
periaqueductal gray, dorsal	

periaqueductal grey	
pituitary gland	
raphe nucleus	
raphe nucleus, dorsal	
raphe nucleus, median	
reticular formation	
septal cerebral peduncle	
septum, dorsolateral	
septum, lateral	
septum, medial	
substantia innominata	
substantia nigra	
substantia nigra - L	
substantia nigra - R	
substantia nigra, reticulata	
thalamus	
thalamus, mediodorsal	
thalamus, parafascicularis nucleus	
thalamus, ventral	
thalamus, ventral posteromedial	
thalamus, ventrolateral	
ventral tegmental area	
ventricle, lateral - R	

Supplementary Table 3. Rat models expressed in Human Protein Atlas and hence included for target prediction analysis.

Uniprot	Name
Q63273	Glutamate receptor ionotropic, kainate 5 (GluK5) (Glutamate receptor KA-2) (KA2)
P35234	Tyrosine-protein phosphatase non-receptor type 5 (EC 3.1.3.48) (Neural-specific protein-tyrosine phosphatase) (Striatum-enriched protein-tyrosine phosphatase) (STEP)
P70606	Small conductance calcium-activated potassium channel protein 1 (SK1) (SKCa 1) (SKCa1) (KCa2.1)
P22771	Glycine receptor subunit alpha-2 (Glycine receptor strychnine-binding subunit)
Q02294	Voltage-dependent N-type calcium channel subunit alpha-1B (Brain calcium channel III) (BIII) (Calcium channel, L type, alpha-1 polypeptide isoform 5) (Voltage-gated calcium channel subunit alpha Cav2.2)
Q00960	Glutamate receptor ionotropic, NMDA 2B (GluN2B) (Glutamate [NMDA] receptor subunit epsilon-2) (N-methyl D-aspartate receptor subtype 2B) (NMDAR2B) (NR2B)
Q00961	Glutamate receptor ionotropic, NMDA 2C (GluN2C) (Glutamate [NMDA] receptor subunit epsilon-3) (N-methyl D-aspartate receptor subtype 2C) (NMDAR2C) (NR2C)
P70579	Metabotropic glutamate receptor 8 (mGluR8)
Q01812	Glutamate receptor ionotropic, kainate 4 (GluK4) (Glutamate receptor KA-1) (KA1)
P62813	Gamma-aminobutyric acid receptor subunit alpha-1 (GABA(A) receptor subunit alpha-1)
P35439	Glutamate receptor ionotropic, NMDA 1 (GluN1) (Glutamate [NMDA] receptor subunit zeta-1) (N-methyl-D-aspartate receptor subunit NR1) (NMD-R1)
P15431	Gamma-aminobutyric acid receptor subunit beta-1 (GABA(A) receptor subunit beta-1)
P42260	Glutamate receptor ionotropic, kainate 2 (GluK2) (Glutamate receptor 6) (GluR-6) (GluR6)
Q63384	Neurotensin receptor type 2 (NT-R-2) (NTR2) (High-affinity levocabastine-sensitive neurotensin receptor)
Q63633	Solute carrier family 12 member 5 (Electroneutral potassium-chloride cotransporter 2) (Furosemide-sensitive K-Cl cotransporter) (K-Cl cotransporter 2) (rKCC2) (Neuronal K-Cl cotransporter)
Q9R1M7	Glutamate receptor ionotropic, NMDA 3A (GluN3A) (Glutamate receptor chi-1) (N-methyl-D-aspartate receptor) (N-methyl-D-aspartate receptor subtype 3A) (NMDAR3A) (NR3A) (NMDAR-L) (NMDAR-L1)
P23978	Sodium- and chloride-dependent GABA transporter 1 (GAT-1) (Solute carrier family 6 member 1)
P18901	D(1A) dopamine receptor (Dopamine D1 receptor)
P09483	Neuronal acetylcholine receptor subunit alpha-4
P68403	Protein kinase C beta type (PKC-B) (PKC-beta) (EC 2.7.11.13)
Q62645	Glutamate receptor ionotropic, NMDA 2D (GluN2D) (Glutamate [NMDA] receptor subunit epsilon-4) (N-methyl D-aspartate receptor subtype 2D) (NMDAR2D) (NR2D)

Q63604	BDNF/NT-3 growth factors receptor (EC 2.7.10.1) (Neurotrophic tyrosine kinase receptor type 2) (TrkB tyrosine kinase) (Trk-B)
O88943	Potassium voltage-gated channel subfamily KQT member 2 (KQT-like 2) (Potassium channel subunit alpha KvLQT2) (Voltage-gated potassium channel subunit Kv7.2)
Q01066	Calcium/calmodulin-dependent 3',5'-cyclic nucleotide phosphodiesterase 1B (Cam-PDE 1B) (EC 3.1.4.17) (63 kDa Cam-PDE)
P70596	Melanocortin receptor 4 (MC4-R)
P04774	Sodium channel protein type 1 subunit alpha (Sodium channel protein brain I subunit alpha) (Sodium channel protein type I subunit alpha) (Voltage-gated sodium channel subunit alpha Nav1.1)
P04775	Sodium channel protein type 2 subunit alpha (Sodium channel protein brain II subunit alpha) (Sodium channel protein type II subunit alpha) (Voltage-gated sodium channel subunit alpha Nav1.2)
Q9QYN8	Histamine H3 receptor (H3R) (HH3R)
P23576	Gamma-aminobutyric acid receptor subunit alpha-2 (GABA(A) receptor subunit alpha-2)
P63138	Gamma-aminobutyric acid receptor subunit beta-2 (GABA(A) receptor subunit beta-2)
P97639	Melanin-concentrating hormone receptor 1 (MCH receptor 1) (MCH-R1) (MCHR-1) (G-protein coupled receptor 24) (MCH-1R) (MCH1R) (MCHR) (SLC-1) (Somatostatin receptor-like protein) (Fragment)
Q62805	Galanin receptor type 1 (GAL1-R) (GALR-1)
P35353	Corticotropin-releasing factor receptor 1 (CRF-R-1) (CRF-R1) (CRFR-1) (Corticotropin-releasing hormone receptor 1) (CRH-R-1) (CRH-R1)
P49187	Mitogen-activated protein kinase 10 (MAP kinase 10) (MAPK 10) (EC 2.7.11.24) (SAPK-beta) (Stress-activated protein kinase JNK3) (c-Jun N-terminal kinase 3) (p54-beta)
Q63010	Liver carboxylesterase B-1 (EC 3.1.1.1) (Liver microsomal carboxylesterase)
P30553	Gastrin/cholecystokinin type B receptor (CCK-B receptor) (CCK-BR) (Cholecystokinin-2 receptor) (CCK2-R)
P12390	Neuronal acetylcholine receptor subunit beta-2 (Neuronal acetylcholine receptor non-alpha-1 chain) (N-alpha 1)
P09216	Protein kinase C epsilon type (EC 2.7.11.13) (nPKC-epsilon)
P09217	Protein kinase C zeta type (EC 2.7.11.13) (nPKC-zeta)
Q9Z0U4	Gamma-aminobutyric acid type B receptor subunit 1 (GABA-B receptor 1) (GABA-B-R1) (GABA-BR1) (GABABR1) (Gb1)
P08413	Calcium/calmodulin-dependent protein kinase type II subunit beta (CaM kinase II subunit beta) (CaMK-II subunit beta) (EC 2.7.11.17)
P35400	Metabotropic glutamate receptor 7 (mGluR7)
P20236	Gamma-aminobutyric acid receptor subunit alpha-3 (GABA(A) receptor subunit alpha-3)
P19969	Gamma-aminobutyric acid receptor subunit alpha-5 (GABA(A) receptor subunit alpha-5)
P35364	5-hydroxytryptamine receptor 5A (5-HT-5A) (5-HT5A) (REC17) (Serotonin receptor 5A)

P46720	Solute carrier organic anion transporter family member 1A1 (Organic anion-transporting polypeptide 1) (OATP-1) (Sodium-independent organic anion transporter 1) (Solute carrier family 21 member 1)
D4A3N4	Adenylate cyclase 1 (Predicted) (Protein Adcy1)
P63319	Protein kinase C gamma type (PKC-gamma) (EC 2.7.11.13)
P25115	D(1B) dopamine receptor (D(5) dopamine receptor) (Dopamine D5 receptor)
P08911	Muscarinic acetylcholine receptor M5
Q01062	cGMP-dependent 3',5'-cyclic phosphodiesterase (EC 3.1.4.17) (Cyclic GMP-stimulated phosphodiesterase) (CGS-PDE) (cGSPDE)
P28646	Somatostatin receptor type 1 (SS-1-R) (SS1-R) (SS1R) (SRIF-2)
Q91ZM7	GABA theta subunit
P23574	Gamma-aminobutyric acid receptor subunit gamma-1 (GABA(A) receptor subunit gamma-1)
P50554	4-aminobutyrate aminotransferase, mitochondrial (EC 2.6.1.19) ((S)-3-amino-2-methylpropionate transaminase) (EC 2.6.1.22) (GABA aminotransferase) (GABA-AT) (Gamma-amino-N-butyrate transaminase) (GABA transaminase) (GABA-T) (L-AIBAT) [Cleaved into: 4-aminobutyrate aminotransferase, brain isoform; 4-aminobutyrate aminotransferase, liver isoform]
P30680	Somatostatin receptor type 2 (SS-2-R) (SS2-R) (SS2R) (SRIF-1)
Q9EPX4	P2Y purinoceptor 12 (P2Y12) (P2Y12 platelet ADP receptor)
P08909	5-hydroxytryptamine receptor 2C (5-HT-2C) (5-HT2C) (5-HTR2C) (5-hydroxytryptamine receptor 1C) (5-HT-1C) (5-HT1C) (Serotonin receptor 2C)
P31647	Sodium- and chloride-dependent GABA transporter 3 (GAT-3) (Solute carrier family 6 member 11)
P31424	Metabotropic glutamate receptor 5 (mGluR5)
P31421	Metabotropic glutamate receptor 2 (mGluR2)
P31422	Metabotropic glutamate receptor 3 (mGluR3)
P31423	Metabotropic glutamate receptor 4 (mGluR4)
Q9R1K8	RAS guanyl-releasing protein 1 (Calcium and DAG-regulated guanine nucleotide exchange factor II) (CalDAG-GEFII) (Ras guanyl-releasing protein)
P63079	Gamma-aminobutyric acid receptor subunit beta-3 (GABA(A) receptor subunit beta-3)
Q00959	Glutamate receptor ionotropic, NMDA 2A (GluN2A) (Glutamate [NMDA] receptor subunit epsilon-1) (N-methyl D-aspartate receptor subtype 2A) (NMDAR2A) (NR2A)
P32301	Glucagon-like peptide 1 receptor (GLP-1 receptor) (GLP-1-R) (GLP-1R)
P42264	Glutamate receptor ionotropic, kainate 3 (GluK3) (Glutamate receptor 7) (GluR-7) (GluR7)
P97612	Fatty-acid amide hydrolase 1 (EC 3.5.1.99) (Anandamide amidohydrolase 1) (Oleamide hydrolase 1)
P34975	Kappa-type opioid receptor (K-OR-1) (KOR-1)
P15823	Alpha-1B adrenergic receptor (Alpha-1B adrenoreceptor) (Alpha-1B adrenoceptor)
P18506	Gamma-aminobutyric acid receptor subunit delta (GABA(A) receptor subunit delta)

P18508	Gamma-aminobutyric acid receptor subunit gamma-2 (GABA(A) receptor subunit gamma-2)
P14842	5-hydroxytryptamine receptor 2A (5-HT-2) (5-HT-2A) (Serotonin receptor 2A)
P35370	Nociceptin receptor (Kappa-type 3 opioid receptor) (KOR-3) (Orphanin FQ receptor) (ROR-C) (XOR1)
P20272	Cannabinoid receptor 1 (CB-R) (CB1) (Brain-type cannabinoid receptor)
P19327	5-hydroxytryptamine receptor 1A (5-HT-1A) (5-HT1A) (Serotonin receptor 1A)
P25099	Adenosine receptor A1
Q09429	ATP-binding cassette sub-family C member 8 (Sulfonylurea receptor 1)
P23385	Metabotropic glutamate receptor 1 (mGluR1)
Q63120	Canalicular multispecific organic anion transporter 1 (ATP-binding cassette sub-family C member 2) (Canalicular multidrug resistance protein) (Multidrug resistance-associated protein 2)
P70605	Small conductance calcium-activated potassium channel protein 3 (SK3) (SKCa 3) (SKCa3) (KCa2.3)
Q9QZN9	Cannabinoid receptor 2 (CB-2) (CB2) (rCB2)
P24942	Excitatory amino acid transporter 1 (Glial glutamate transporter) (Sodium-dependent glutamate/aspartate transporter 1) (GLAST-1) (Solute carrier family 1 member 3)
P08104	Sodium channel protein type 3 subunit alpha (Sodium channel protein brain III subunit alpha) (Sodium channel protein type III subunit alpha) (Voltage-gated sodium channel subtype III) (Voltage-gated sodium channel subunit alpha Nav1.3)
Q924U1	KiSS-1 receptor (KiSS-1R) (G-protein coupled receptor 54) (G-protein coupled receptor OT7T175) (rOT7T175) (Kisspeptins receptor) (Metastin receptor)
P08482	Muscarinic acetylcholine receptor M1
P08483	Muscarinic acetylcholine receptor M3
P08485	Muscarinic acetylcholine receptor M4
P19493	Glutamate receptor 4 (GluR-4) (GluR4) (AMPA-selective glutamate receptor 4) (GluR-D) (Glutamate receptor ionotropic, AMPA 4) (GluA4)
P19492	Glutamate receptor 3 (GluR-3) (AMPA-selective glutamate receptor 3) (GluR-C) (GluR-K3) (Glutamate receptor ionotropic, AMPA 3) (GluA3)
P19491	Glutamate receptor 2 (GluR-2) (AMPA-selective glutamate receptor 2) (GluR-B) (GluR-K2) (Glutamate receptor ionotropic, AMPA 2) (GluA2)
P19490	Glutamate receptor 1 (GluR-1) (AMPA-selective glutamate receptor 1) (GluR-A) (GluR-K1) (Glutamate receptor ionotropic, AMPA 1) (GluA1)
P22756	Glutamate receptor ionotropic, kainate 1 (GluK1) (Glutamate receptor 5) (GluR-5) (GluR5)
Q9JJ16	5-hydroxytryptamine receptor 3B (5-HT3-B) (5-HT3B) (Serotonin receptor 3B)
O88871	Gamma-aminobutyric acid type B receptor subunit 2 (GABA-B receptor 2) (GABA-B-R2) (GABA-BR2) (GABABR2) (Gb2) (G-protein coupled receptor 51)
P33533	Delta-type opioid receptor (D-OR-1) (DOR-1) (Opioid receptor A)
P28471	Gamma-aminobutyric acid receptor subunit alpha-4 (GABA(A) receptor subunit alpha-4)

Q9ERC0	Neuropeptide Y/peptide YY-Y2 receptor (Protein Npy2r) (RCG62488)
P11275	Calcium/calmodulin-dependent protein kinase type II subunit alpha (CaM kinase II subunit alpha) (CaMK-II subunit alpha) (EC 2.7.11.17)
