# Supplementary material for: Systemic neurotransmitter responses to clinically approved and experimental neuropsychiatric drugs
Source: Nat Commun. 2018 Nov 8;9:4699. doi: 10.1038/s41467-018-07239-1 (PMC6224407; doi:10.1038/s41467-018-07239-1)
Supplement: Supplementary file 3 — Description of Additional Supplementary Files [file 41467_2018_7239_MOESM3_ESM.docx]

Description of Additional Supplementary Files

**Dataset 1:** Data included in syphad database represented as a 10,511x18 matrix.
